# Redox Shift from Antioxidant to Pro-Oxidant Activity Induced by Nanoencapsulated α-tocopherol in Diets for Shrimp (*Litopenaeus vannamei)*

**DOI:** 10.1007/s10126-026-10609-2

**Published:** 2026-04-24

**Authors:** Andrea Manriquez-Patiño, Adrián Ríos-Ortiz, Rafael Vázquez-Duhalt, Rocío A. Chávez-Santoscoy, Aurora Tinajero, María Teresa Viana

**Affiliations:** 1 Doctorado en Oceanografía Costera, Facultad de Ciencias Marinas, Universidad Autónoma de Baja California (UABC), Baja California (BC), Km 107 carretera Tij/Eda, Ensenada, 22860 México; 2 Doctorado en Medio Ambiente y Desarrollo, Instituto de Investigaciones Oceanológicas (IIO), UABC, Ensenada, BC México; 3 Centro de Nanociencias y Nanotecnología, Universidad Nacional Autónoma de México, Ensenada, BC México; 4 Tecnológico de Monterrey, Escuela de Ingeniería y Ciencias, Ave. Eugenio Garza Sada 2501 Sur, Col: Tecnológico, 64700 Monterrey, N.L México; 5 IIO, UABC, Ensenada, BC México

**Keywords:** Peroxidation, Additives, Stress, Gene expression, Feed

## Abstract

Shrimp farming has seen significant growth in recent years, with increased production leading to intensification that requires less area to achieve higher yields. Intensive shrimp farming increases susceptibility to oxidative stress due to hypoxia and thermal fluctuations, compromising productivity and survival. Although α-tocopherol is widely used as a dietary antioxidant, its bioavailability and stability are limited along the intestinal tract. In this study, α-tocopherol was nanoencapsulated in chitosan via ionic gelation to enhance systemic delivery of α-tocopherol in *Litopenaeus vannamei*, resulting in nanoparticles with a diameter of 150 nanometers. For two weeks, four dietary treatments with five replicates were fed different nanoencapsulated α-tocopherol levels, (0, 2, 3, and 4 mg kg^-1^). A system of 20-aquariums was used, each aquarium defined as the experimental unit (EU). Prior to the experiment, nanoparticles labeled with fluorescent FITC were used to confirm whether they crossed the intestinal barrier. Gene expression analysis revealed a dose-dependent catalase (CAT) modulation of antioxidant enzymes, such as superoxide dismutase (MnSOD), glutathione peroxidase (GPX), and glutathione S-transferase (GST) in the hepatopancreas, with transcriptional downregulation (qRT-PCR) at higher concentrations, suggesting reduced oxidative pressure or a shift toward pro-oxidant signaling. Despite the short length of the experimental procedure, these findings suggest that nanoencapsulated α-tocopherol not only enhances delivery efficiency but also unveils a redox transition threshold, highlighting the dual antioxidant/pro-oxidant nature of α-tocopherol in vivo. Nanotechnology with biomaterials such as chitosan presents a promising approach to mitigate oxidative stress by enhancing the stability and release of essential antioxidants. Furthermore, this work provides mechanistic insight into nanonutraceutical strategies for oxidative stress management in aquaculture. The pro-oxidant shift under longer experimental procedures is discussed.

## Introduction

Mexico is the second-largest shrimp producer in Latin America. In 2024, it generated 273,900 MT (CONAPESCA, 2025). Therefore, technological innovations are currently being sought to improve its competitiveness and sustainability. However, increasing shrimp production requires intensifying farming practices (e.g., increasing production density). However, a lack of space, low oxygen levels (hypoxia), and rising water temperatures in farms increase oxidative stress, which in turn reduces growth, immune capacity, and health, thereby increasing the likelihood of disease outbreaks (Bao et al. 2018; Breitburg et al. 2018).

In crustaceans, as in other organisms, the loss of homeostasis is reflected in gene expression. For this reason, it is essential to investigate how feed additives can impact these changes, aiding in restoring homeostasis or potentially impairing health status as prooxidants. Stress harms growth and, consequently, decreases productivity (Díaz et al. 2013). According to Ren et al. (2021), the categories of responses to environmental stress are divided into three sections: primary (e.g., the release of corticosteroids and catecholamines and the neuroendocrine response), secondary (e.g., immunological, osmoregulatory, hematological, cellular, and metabolic changes), and tertiary (e.g., behavioral changes and physiological responses to stress throughout the organism). One consequence of organisms being exposed to environmental stress is the generation of oxidative stress, which is caused by an excess of reactive oxygen species (ROS) and/or a reduction in antioxidant systems for handling ROS. These reactive species can be produced by different sources, for example, from the activity of soluble oxidase enzymes, nitric oxide synthase, and NADPH oxidase at the phagocyte membrane. ROS, such as hydrogen peroxide (H_2_O_2_), the superoxide anion (·O_2_−), and the hydroxyl free radical (·OH), may be involved in cellular aging and various inflammatory disorders, as well as in efficiently combating pathogens, including bacteria and viruses. They also play an important role in immune signal transduction. Additionally, they are responsible for modifications in glycoprotein carbohydrates, which can even cause DNA strand breaks and base modifications through methylation (Parrilla-Taylor et al. 2013).

In crustaceans such as the white shrimp *Litopenaeus vannamei*, the expression of antioxidant enzymes is one of the first responses to protect against oxidative stress damage. These include glutathione peroxidase (GPx), superoxide dismutase (SOD), and catalase (CAT) (Duan et al., 2015). SOD scavenges superoxide anions, converting them into hydrogen peroxide and oxygen. Hydrogen peroxide is then transformed into water and oxygen through CAT or GSH-Px. Therefore, enhancing antioxidant capacity is crucial for aquatic animals to overcome environmental stress and maintain their productivity (Wang et al., 2015).

The use of feed additives, as well as functional feeds, has been used for many years and across various species (Bautista et al. 1992). For this purpose, various products have been evaluated that can mitigate oxidative stress, which, ultimately, whether due to temperature or hypoxia, is expressed similarly in shrimp (Estrada-Cárdenas et al., 2021). At the same time, a large number of antioxidants are recommended for stress alleviation, such as vitamin E (α-tocopherol), as well as synthetic products like butylated hydroxytoluene (BHT), and even molecules described as phytobiotics. Vitamin E is fat-soluble and found in eight different forms, of which α-tocopherol (α-Toc) is the main radical-scavenging antioxidant in vivo. It effectively interrupts the chain of lipid oxidation, thereby protecting polyunsaturated fatty acids and low-density lipoproteins from oxidation (Aresta et al., 2014). However, when provided in excess, they can also interfere with natural processes that require a certain level of free radicals to function correctly and can even lead to toxicity. According to Swift et al. (2014), severe dermatological inflammation was observed in mice after injection of 800 mg Kg^−1^ of α-tocopherol, with signs of hepatotoxicity. On the contrary, Kappus and Diplock (1992) reported that even high doses of oral vitamin E did not produce adverse effects, possibly neutralized through the intestinal tract. Therefore, to study antioxidant metabolism, it is essential to ensure that vitamin E, as well as any other antioxidant, is absorbed through the intestinal barrier to study in detail the effect of antioxidants on metabolism.

When antioxidants are added to food, they can be easily neutralized upon contact with dietary ingredients, during storage (shelf life), and in the intestine during the digestive process, and do not necessarily enter the body through the enterocytes. Therefore, their primary benefit is to prevent food rancidity, without providing a direct physiological benefit to organisms (Bautista et al. 1992).

Polymeric nanovehicles have emerged as efficient platforms for the protection and controlled delivery of bioactive compounds, particularly those with limited stability and bioavailability. Among them, chitosan-based nanoparticles have been extensively used for encapsulating therapeutic proteins, enzymes, and other bioactive molecules due to their biocompatibility, biodegradability, and low toxicity (Racovita et al., 2009; Quester et al. 2022). The application of nanoparticles has been widely proposed, the structural characteristics of the chitosan polymeric matrix provide a protective shield, improving the thermo-oxidative stability of encapsulated compounds (Wu et al. 2020). Moreover, they protect encapsulated compounds during processing, storage, and gastrointestinal transit, which is particularly relevant in stomach-less aquatic organisms. These characteristics make chitosan nanoparticles attractive candidates for nutraceutical applications in aquaculture. Therefore, we hypothesized that chitosan nanoencapsulation would effectively enhance the systemic delivery of α-tocopherol in *Litopenaeus vannamei*, providing antioxidant protection. To test this theory, the present study evaluates the systemic delivery of these nanoparticles and assesses their uptake via fluorescein isothiocyanate (FITC) labeling to trace internalization. Additionally, shrimp were fed graded dietary concentrations of α-tocopherol-loaded nanoparticles to determine their physiological effects. Specifically, this study investigates reactive oxygen species (ROS) generation and the transcriptional modulation of key antioxidant enzymes to identify the exact dose of this potential antioxidant/pro-oxidant shift.

## Materials and Methods

### Synthesis of Chitosan Nanoparticles

The synthesis of nanocapsules was performed according to the methodology of Quester et al. (2022). In summary, a 0.25% chitosan solution was prepared by mixing 0.25 g of chitin with 100 mL of 2% acetic acid. The mixture was stirred for 48 h or until it was completely homogeneous. It was then centrifuged at 8000 × *g* for 15 min at 4 °C, followed by centrifugation at 11,000 × *g*. The resulting chitosan solution (5 mL) was stirred at 800 rpm for 10 min. While stirring, 200 µL of an α-tocopherol solution dissolved in a 1:1 DMSO/water mixture at a concentration of 100 µM was added to load the nanoparticles with α-tocopherol (Ch-NPs-Toc). The Ch-NPs-Toc were formed by ionic gelation by adding 1 mL of sodium tripolyphosphate (0.025%) at a rate of 0.09 mm/s using an automated injection-controlled drip system (Poseidon software drop system) through a 3 mL syringe (8.66 mm internal diameter) and stirring for 1 h. The nanoparticle preparation was crosslinked by adding 100 µL of glutaraldehyde (2.5%) at a rate of 0.09 mm/s through a 1 mL syringe (4.6 mm i.d.) and left to stir for 1 h. The non-reacted aldehyde groups were neutralized by adding 1 mL of 2 mM glutamine (Gallardo et al. 2019). To remove free and unencapsulated chitosan, α-tocopherol, and FITC, the solutions were centrifuged at 16,000 × g for 30 min at 4 °C using a Sorvall Legend XT rotor (Thermo Scientific, 73006479). The supernatants were subsequently centrifuged at 26,000 × g for 1 h at 4 °C in a refrigerated microcentrifuge (Sorvall Legend Micro 17R rotor, Thermo Scientific).

To monitor the Ch-NPs, the nanoparticles were labeled with a fluorophore, fluorescein isothiocyanate (FITC). A solution of 50 µL containing 5 mg of FITC dissolved in 1 mL of absolute ethanol was added dropwise using an insulin syringe (1 mL) to the chitosan solution before nanoparticle formation.

### Nanoparticle Characterization

#### Dynamic Light Scattering (DLS)

Nanoparticle characterization was performed by measuring the hydrodynamic diameter using dynamic light scattering (DLS) and determining the zeta potential using a Zetasizer nano ZS90 instrument (Malvern, UK). The effective surface charge was estimated as Z potential in a buffer of pH 6.8.

#### Transmission Electron Microscopy (TEM)

Newly synthesized chitosan nanoparticles were characterized by morphological and surface topographic analysis using transmission electron microscopy (TEM) in a JEOL ARM-200 F with spherical aberration corrector, operated at 200 keV, and equipped with an EDS Oxford AZTecTEM detector.

#### Encapsulation Efficiency

A standard curve for α-tocopherol was obtained using UV-Vis spectrophotometry. Initially, a primary stock solution was formulated by dissolving α-tocopherol in methanol to achieve a concentration of 5 mg mL^−1^. Then it was serially diluted with high-purity methanol to yield a set of working standards with concentrations ranging from 5 to 75 µg mL^−1^. Absorbance measurements were performed using a VarioSkan Lux microplate spectrophotometer. Aliquots of 300 µL of each standard solution were loaded in triplicate into a 96-well plate (Brand F). Methanol was used as the blank. The absorption spectrum of each standard was scanned from 250 nm to 350 nm. A characteristic absorption maximum was identified at 290 nm for all α-tocopherol samples. The absorbance values at this wavelength were recorded and plotted against their respective concentrations to generate the standard curve. The relationship between absorbance and concentration was subsequently determined through linear regression analysis.

The validated spectrophotometric method was applied to determine the encapsulation efficiency (EE) of α-tocopherol within chitosan-based nanoparticles (Ch-NPs-Toc). For the analysis, 500 µL aliquots of the nanoparticle suspensions were subjected to an acid hydrolysis step by the addition of a drop of concentrated hydrochloric acid, effectively disrupting the nanocapsules integrity. The released α-tocopherol was then extracted into an organic phase via three successive washes with 500 µL of dichloromethane, each involving 5 min of vigorous mixing. The combined dichloromethane extracts, containing the target compound, were collected. The solvent was gently evaporated under a stream of nitrogen gas. The resulting dry residue, comprising the extracted α-tocopherol, was re-dissolved in 1 mL of methanol. The absorbance of this final solution was measured at 290 nm.

The mass of α-tocopherol recovered from the nanoparticles was calculated by interpolating the absorbance reading onto the pre-constructed standard curve. The encapsulation efficiency (EE), expressed as a percentage, was then calculated using the following formula:

1 $$\:EE\left(\%\right)=\frac{Initial\:amount\:of\:utilized\:solution}{Amount\:found\:in\:nanoencapsules}\times100$$

#### Release

Since α-tocopherol is a strong hydrophobic substance, its release was evaluated using cod liver oil as a lipophilic matrix. For the release assay, 350 µL of the Ch-NPs-Toc suspension was introduced into 650 µL of cod liver oil, and the mixture was subjected to continuous agitation in triplicate samples. The release profile was assessed at two time points: 2 h and 24 h. Following each incubation period, the solutions were centrifuged at 3000 rpm for 30 min to achieve phase separation. The resulting product was recovered, yielding two distinct phases: a polar phase (containing chitosan) and a non-polar phase (containing the cod liver oil with the released compound). The content of α-tocopherol within the non-polar cod liver oil phase was quantified directly using UV-Vis spectroscopy by measuring the absorbance at 290 nm. The concentration of the compounds was determined by interpolation from a pre-established calibration curve, which was constructed using cod liver oil as the solvent to account for matrix effects.

To determine the fraction of α-tocopherol that remained encapsulated within the nanoparticles in the aqueous phase, the polar phase was subsequently analyzed. The residual compounds were extracted via acid hydrolysis using hydrochloric acid, followed by liquid-liquid extraction with dichloromethane, in accordance with the same protocol previously described for determining encapsulation efficiency.

#### Antioxidant Activity

To assess the nanoparticles’ antioxidant activity, an ABTS•⁺ radical cation solution was prepared according to the method of Chanput et al. (2016) and expressed as Trolox Equivalents (TE). The assay was performed by adding 5 µL of each standard solution to 250 µL of the ABTS•⁺ solution (7 mM) in a microplate well. Following a 5-minute incubation in the dark, absorbance was measured with a VarioSkan Lux (Thermo Fisher Scientific) microplate reader, and all analyses were conducted in triplicate. To compare the antioxidant capacity, samples of α-tocopherol—both in its free form and chitosan-encapsulated—were diluted in methanol to 298 µg mL^−1^, matching the oil content in the nanoparticle formulations. The assay for these samples was carried out identically to the calibration procedure, using 5 µL of sample, and was also performed in triplicate.

### Experimental Design and Feed Preparation

For the nanoparticles labelled with FITC, the experimental unit consisted in six conical tanks (350 L), divided into two groups: three control tanks without FITC and three experimental tanks supplemented with a diet containing nanoparticles loaded with FITC. Each treatment was performed in triplicate, and each tank contained five shrimp (30 in total) with an average weight of 6–8 g. The feeding phase began with the administration of diets in complete darkness since the FITC is sensitive to light. After three days of feeding, one shrimp from each tank was sacrificed, and hepatopancreas samples were collected for analysis. Furthermore, all treatments were fed with the control diet for an additional ten days. At the end of this period (day 13), a second sampling of hepatopancreas tissue was performed using the same procedure.

For the α-tocopherol nanoparticles experiment, a 25-aquarium system was used (20 L). The experimental unit (EU) was defined as the individual aquarium, resulting in four treatments in quintuplicate (*n* = 5 replicate aquaria per treatment). To minimize stress due to dominance and isolate the physiological response from behavior, each EU had only one shrimp, which was randomly allocated to each container. All aquaria were connected to a recirculating aquaculture system (RAS), each one equipped with an airstone and maintained at a constant temperature. Oxygen, salinity, and temperature levels in the aquariums were measured daily using a YSI-55 (YSI Inc., Yellow Springs, OH, USA). Total nitrite and ammonium levels were measured twice weekly using API test kits (Mars Fishcare Inc., Chalfont, PA, USA). The temperature was maintained at 28 °C, and the salinity ranged from 34 to 35 PSU, with an oxygen level of 6–8 mg L^−1^. One shrimp (*L. vannamei*) was randomly added to each EU. Shrimp were obtained from the FitMar commercial laboratory in Mazatlán, Sinaloa, Mexico, and fed in excess, but according to their size (10–11 g each). Diets were formulated in blocks of five different diets, each containing the appropriate shrimp nutrients and corresponding additives (Table 1). The feed was prepared in the Nutrition and Digestive Physiology Laboratory (FEED-AQUA Laboratory and LINDEAACUA Laboratory) at the Ensenada campus of the Autonomous University of Baja California (UABC), according to established protocols. Briefly, the macro ingredients were pulverized, sieved, and mixed in a vertical chopper/mixer until a homogeneous mass was obtained. The micro ingredients (including each additive) were mixed and incorporated into the mixture along with the liquid ingredients (gelatin and oils), blending until the desired texture was achieved. Then, the diets were formed using a syringe to expel the feed mixture, resulting in a diameter of 1 mm. The strips were then cut to 3 mm and stored frozen in individual packages (approx. 1 g each).

**Table 1:** Ingredient composition (%) on a dry matter basis of five diets containing four levels of nanoencapsulated α-tocopherol for shrimp (*Litopenaeus vannamei*) for 15 days

Ingredient Composition (%)	Dietary Treatments			
	Control	NPsTc2	NPsTc3	NPsTc4
Fish meal (65% CP) ^a^	80	80	80	80
Gelatin ^b^	10	10	10	10
Corn Starch ^c^	5	5	5	5
Fish oil ^d^	5	5	5	5
NPs α-tocopherol (mg Kg⁻¹)	-	0.002	0.003	0.004
Total	100	100	100	100
***Proximate composition (in percentage)***				
Crude protein	40.0	40.0	40.0	40.0
Crude fat	9.0	9.0	9.0	9.0

^a^ Sardin meal from Baja Marin, Ensenada BC, México

^b^ Progel Mexicana S.A. de C.V., León, Guanajuato, México; Ingredión México S.A. de C.V

^c^ Maizena™

^d^ From Sardin (Baja Marin, Enenada BC, México)

### RNA Extraction and cDNA Synthesis

Hepatopancreas tissue samples (≈35 mg) stored in RNAlater (Ambion) were homogenized using a micropistillator. RNA extraction was performed individually using the PureLink^®^ RNAlink Mini Kit (Ambion). RNA integrity was measured by electrophoresis on a 1% agarose gel stained with 10x red dye (Biotium Inc., Hayward, CA). Concentration and purity were measured using a spectrophotometer (Nanodrop^®^ Lite, Thermo Fisher Scientific Inc., Wilmington, USA). RNA samples with OD_260 nm_–OD_280 nm_ ratios between 1.90 and 2.10 were used for expression quantification. Genomic DNA (gDNA) was extracted with PureLink^®^ DNase (Invitrogen) following the manufacturer’s instructions. The absence of DNA in the samples was confirmed by 1.5% agarose gel electrophoresis and PCR, using rpl8 as a control for amplification. Reverse transcription of total RNA (1 µg) was performed in a 20 µL reaction vessel using the High-Capacity cDNA Reverse Transcription Kit (Applied Biosystems; Carlsbad, CA, USA) in a 96-well Veriti thermal cycler (Applied Biosystems). The program specifications consisted of 10 min at 25 °C, 120 min at 37 °C, and 5 min at 85 °C, followed by a final hold at 4 °C.

### Quantitative Real Time qPCR

The qRT-PCR reactions were performed with 1 ng of sense and antisense cDNA primers (200 nM each, indicated in Table 2) and SYBR^®^ Select Master Mix (Applied Biosystems). Reactions were carried out in 10 µL MicroAmp^®^ Fast Optical 96-well reaction plates (Applied Biosystems) covered with MicroAmp^®^ Optical Adhesive Film (Applied Biosystems). The primers used in this study were selected from López-Galindo et al. (2023) based on the mRNA sequences available in the GenBank database of the National Center for Biotechnology Information (NCBI).

**Table 2:** Access registration numbers and primer sequences for qPCR

Gene	GenBank Accession no.	Forward sequence (5’−3’)	Reverse sequence (5’−3’)	Amplicon length (bp)	eff	
*LvGAPDH*	MG787341.1	TCGGCAAGGAGTGCTCTTAT	GCCTTAGCGTCAAAGATGGA	150	2.10	*
*LvEf1α*	GU136229	GAAATCCGACAACATGGGCT	CCAATCTTGTACACGTCCTG	162	1.97	López-Galindo et al. (2023)
*LvrpL8*	DQ316258	ATGAACCCTGTAGAGCATCCTC	CTTTGTACCACGGATGAGACCA	141	2.09	López-Galindo et al. (2023)
*LVcMnSOD*	DQ029053	CGTAGAGGGTATTGTCGTACAG	AGGGCGTTGAAATCATACTTGAG	152	1.98	*
*LVCat*	AY518322	GCGACCAGAAACAACACACC	CTTGATGCCTTGGTCCGTCT	165	1.91	*
*LVGPx*	AY973252.2	GGACTTCCACCAGATGAACC	CTCGAAGTTGTTCCCAGGAC	153	1.99	López-Galindo et al. (2023)
*LV GST*	A0A3R7NV20	ACACGGAGGAGACCATCGAC	CGACAACCTCCATCTGCCGA	195	1.84	*

bp: base pairs; eff: primer efficiency. *Primers designed in Primer3 software for this study

In addition to the target genes, three potential reference genes were included: elongation factor 1-alpha (EF1α), ribosomal protein L8 (rpl8), and glyceraldehyde-3-phosphate dehydrogenase (GAPDH). Stability analyses were performed using the RefFinder platform, and rpl8 was used as a reference gene according its efficiency calculated from cDNA with serial dilutions (1:10).

PCR conditions were as follows: an initial denaturation step and polymerase activation for 10 min at 95 °C; 40 cycles of denaturation for 15 s at 95 °C, harvesting and extension for 45 s at 60 °C; and a final melting curve from 60 to 95 °C for 20 min to verify primer dimer artifacts. Relative expression quantification was calculated using the ΔΔCT method (Hellemans et al. 2007), using an automated threshold and a moving baseline to determine CT values. All normalized relative expression quantities were log-transformed for graphical representation (Zar 1999).

### Statistical Analyses

Given the experimental design of one shrimp per aquarium, the individual shrimp (strictly corresponding to the aquarium) was defined as the EU with five repetitions, and the individual data from the five replicates were used for the statistics. Normality was tested with the Shapiro-Wilk W test and homogeneity of variance with Levene’s test. Statistical tests were performed using STATISTICA^®^ software (StatSoft, Inc., USA), employing a one-way analysis of variance (ANOVA), with a significance level of *P* ≤ 0.05. In cases where significant differences between means are found, Tukey’s post hoc test was used. Linear or polynomial regressions will also be adjusted, using the same significance scale.

## Results

### Nanoparticle Characterization

**Fig. 1:**
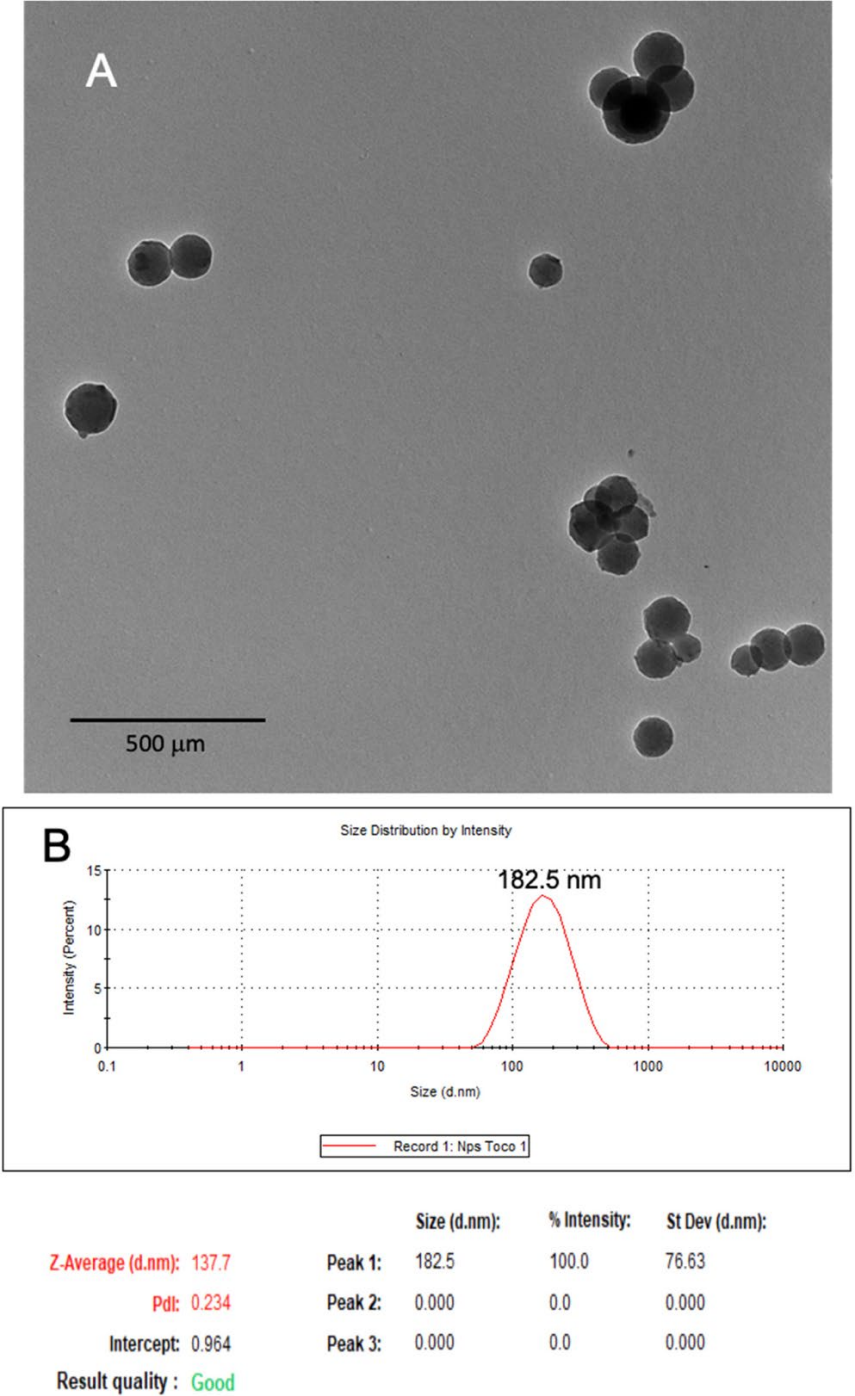
(**A**) Image from transmission electron microscopy (TEM) showing the nanoparticles’ morphology, and (**B**) nanoparticles’ size determined by dynamic light scattering (DLS) analysis

Chitosan nanoparticles containing tocopherol (Ch-NPs-Toc) were synthesized. Their size distribution was characterized using dynamic light scattering (DLS), which showed a mean hydrodynamic diameter of 182.5 ± 76.6 nm and a zeta potential of 52.5 ± 2.5 mV, and a polydispersity index (PDI) of 0.234. In addition, images from TEM were obtained showing semispherical nanoparticles and sizes consistent with the hydrodynamic diameters obtained by DLS (Fig. 1). The absorbance spectra of α-tocopherol (α-Toc) showed a maximum absorbance at 290 nm, which is consistent with previously reported data (Gamze,^ 2020^).

To determine the α-tocopherol loading capacity of the nanoparticles, a calibration curve was obtained using spectrophotometry, yielding a linear regression with a correlation coefficient (R²) of 0.99. The chitosan-based nanoparticles (Ch-NPs-Toc) exhibited an encapsulation efficiency (EE) of 20% ±0.02, which is a sufficient amount to perform the in vivo studies.

**Fig. 2:**
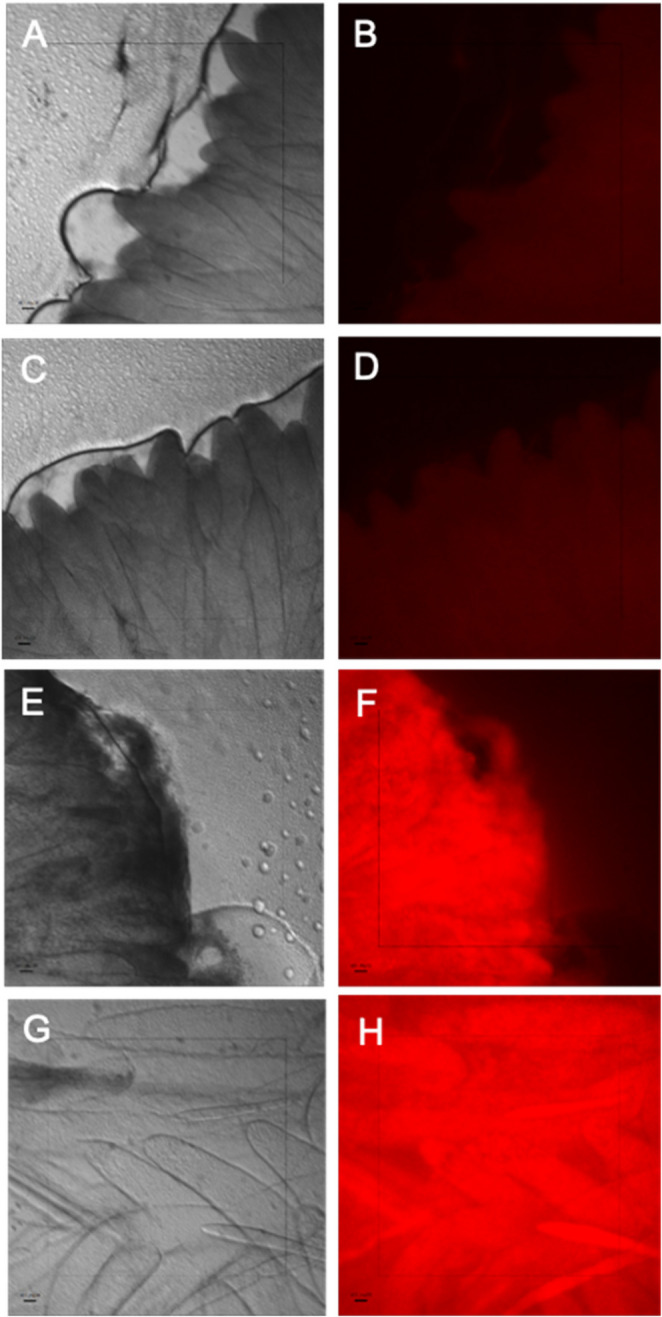
Fluorescence microscopy images of hepatopancreas sections from shrimp. Brightfield images (**A**, **C**, **E**, and **G**) and red fluorescence (**B**, **D**, **F**, and **H**). Control Experiments without nanoparticles (**A**, **B**, **C**, and **D**) and treatment with nanoparticles after 3 days (**E** and **F**) and after 13 days (**G** and **H**)

To monitor the distribution of chitosan nanoparticles inside the hepatopancreas, the nanoparticles were labeled with the fluorophore FITC and observed using a fluorescence microscope. Figure 2 clearly shows that intense red fluorescence is homogeneously distributed in the hepatopancreas of organisms treated with chitosan nanoparticles. While the control experiments without nanoparticle treatment showed a slight autofluorescence.

The antioxidant capacity of Ch-NPs-Toc was determined by the ABTS (2,2’-azino-bis (3-ethylbenzothiazoline-6-sulfonic acid)) assay. The antioxidant compounds have the ability to scavenge the stable ABTS radical cation (ABTS•⁺). The antioxidant activity of α-tocopherol, in both its free and nanoencapsulated forms, showed a marked difference.

Free α-tocopherol demonstrated an antioxidant activity of 148.9 mg TE g^−1^ sample, whereas the nanoencapsulated α-tocopherol exhibited a significantly higher activity of 341.7 mg TE g^−1^ sample. This could be due to the protection of the α-tocopherol molecule when it is encapsulated and the chitosan antioxidant capacity (Yen et al. 2008).

Due to the possibility that α-tocopherol may be partitioned from nanoparticles into tissue fats, an experiment in which Ch-NPs-Toc were exposed to cod liver oil for 2 and 24 h was carried out. The amount of α-tocopherol in the oily phase was spectrophotometrically measured. No α-tocopherol could be detected in the hydrophobic phase, indicating that the compound was not released from the nanoparticles. On the contrary, all α-tocopherol was detected in the aqueous phase containing the Ch-NPs-Toc. This experiment was performed in triplicate.

### Expression of Antioxidant Enzyme Genes in Shrimp

**Fig. 3:**
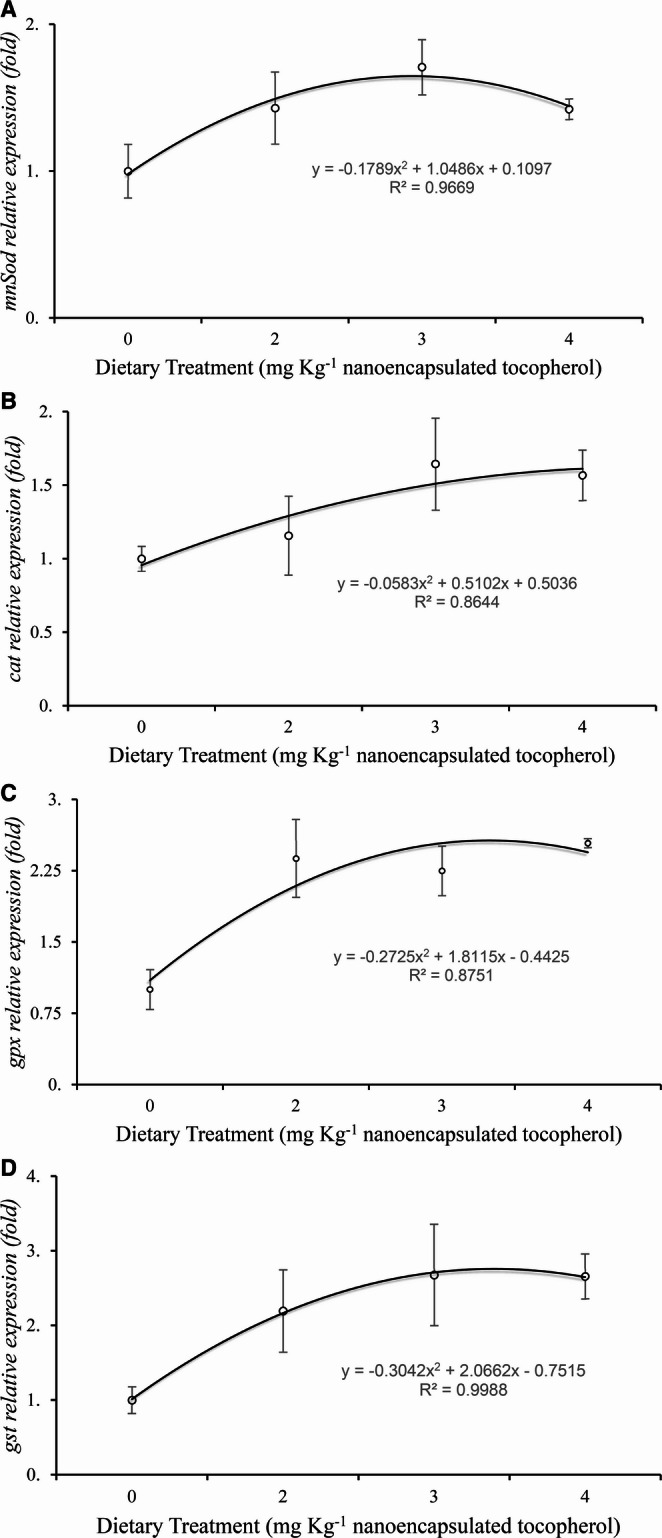
Relative expression of (**A**) manganese superoxide dismutase (*MnSod)*, (**B**) catalase (*cat)*, (**C**) glutathione peroxidase (*gpx)*, and (**D**) glutathione-S-transferase (*gst)* in hepatopancreas of shrimp fed with different levels of nanoencapsulated tocopherol. Data are expressed as fold change relative to the control group (0 mg Kg^−1^), which was set to 1. Error bars represent standard deviation

The transcriptional profiles of four key antioxidant enzymes: manganese superoxide dismutase (MnSOD), catalase (CAT), glutathione peroxidase (GPX), and glutathione S-transferase (GST) (Fig. 3), revealed that dietary supplementation with nanoencapsulated α-tocopherol significantly modulated the relative expression of the *MnSod*,* gst*,* gpx*, and *cat* in shrimp exposed to four treatments (0, 2, 3, and 4 mg Kg^−1^; *n* = 5). The expression levels of the target genes in the experimental groups are presented as fold changes relative to the control group (mg Kg^−1^), which was normalized to a baseline value of 1. In all cases, the response exhibited a quadratic dose-dependent pattern, characterized by an initial upregulation at lower to intermediate doses, followed by attenuation or downregulation at the highest concentration.

Regarding the GPX expression, a maximum level was observed at T1 (2 mg kg^−1^), with an increase of approximately 30% compared to the control group. This upregulation was followed by a moderate decline at T2 and a marked reduction at T3, suggesting a threshold-dependent modulation. The GST expression showed a similar trend, but with a peak shifted towards T2 (3 mg Kg^−1^), resulting in an approximately 80% increase compared to the control. *MnSod* expression showed its most pronounced upregulation at T2, exceeding 70% above control levels, indicating enhanced superoxide detoxification at intermediate supplementation. In contrast, the CAT showed a progressive increase from the control to T2, remaining stable at T3. Unlike the other enzymes, the CAT response was less pronounced.

**Fig. 4:**
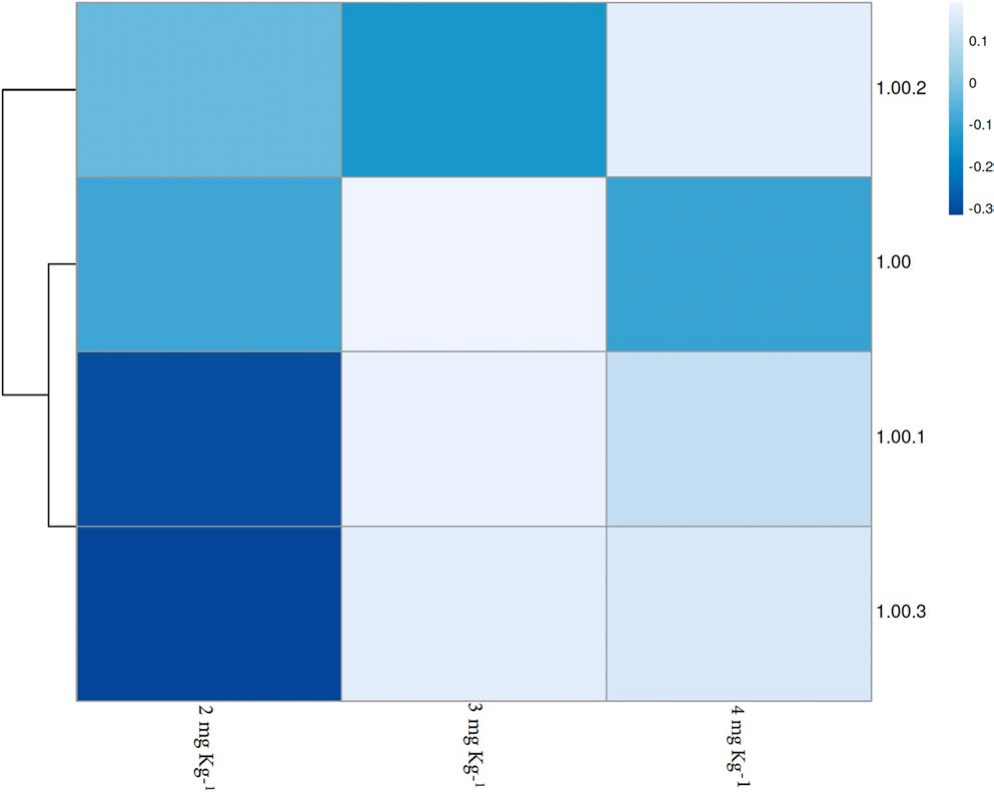
Heatmap illustrating the relative expression (fold change) of antioxidant-related genes (mnSod, cat, gpx, and gst) in the hepatopancreas of *Litopenaeus vannamei* fed varying concentrations of nanoencapsulated α-tocopherol (0, 2, 3, and 4 mg kg⁻¹). The color scale represents the fold change values, where warmer colors indicate higher expression levels (upregulation) and cooler colors indicate baseline or lower expression levels relative to the control group (0 mg kg⁻¹)

Furthermore, a multivariate heatmap visualization (Fig. 4) confirmed the holistic, dose-dependent activation of the antioxidant defense system, clearly highlighting the coordinated peak expression of mnSod, cat, gpx, and gst at the 2 and 3 mg kg⁻¹ inclusion levels.

Altogether, these results indicate a dose-dependent redox regulatory response to nanoencapsulated α-tocopherol, with intermediate concentrations promoting transcriptional activation of antioxidant defenses, whereas higher doses attenuate this response.

## Discussion

Chitosan nanocapsules of 182.5 ± 76.6 nm diameter and containing 20% of α-tocopherol were effectively obtained. This loading efficiency was sufficient to ensure effective in vivo supplementation. It is possible to obtain better loadings by covalently conjugating chitosan with tocopherol and using membrane emulsification, where an encapsulation rate of almost 100% can be achieved (Trombino et al. 2022). In contrast, the nanoencapsulation approach used here preserves release potential while enhancing compound stability. Nevertheless, the observed enhancement in antioxidant efficacy is likely because of the nanoencapsulation process. The chitosan-based nanoparticles may function as a protective barrier, shielding the α-tocopherol from degradation. Furthermore, the intrinsic antioxidant properties of chitosan (Yen et al. 2008) may contribute to the synergistic redox-modulating effect observed in this work.

It is known that vitamin E has been categorized as a primary fat-soluble antioxidant whose primary function is to stop the propagation of lipid peroxidation (Lee and Han 2018). According to Lee and Shiau (2004), shrimp require a level of 85–89 mg Kg^−1^ of vitamin E in their feed to maximize growth and improve their immune response. However, Fernandez-Gimenez et al. (2004) note that the reqirements may vary when other antioxidant factors are present and emphasize the importance of using high-quality oils, as this reduces the need for vitamin E supplementation. During the manufacture and storage of the feed, the oils in the feed can oxidize due to the use of low-quality ingredients or improper storage conditions, such as high temperatures. Therefore, the use of synthetic chemical antioxidants, such as BHT, could be added to reduce the cost impact of vitamin E. In these cases, part of the vitamin E acts as an antioxidant on the fats in the feed, which is why they report that the amount of vitamin E depends on the polyunsaturated fat composition of the diet. Other studies on shrimp have demonstrated that 70 g Kg^−1^of dietary lipids, combined with 1 g Kg^−1^of vitamin C and 0.3 g Kg^−1^of vitamin E, conclude that higher amounts of dietary lipids increase the requirements for vitamins C and E as antioxidants (Ebadi et al. 2021). Therefore, our study aimed to nanoencapsulate alpha-tocopherol (the most active form of vitamin E), preventing vitamin E from acting as an antioxidant on fats in the feed, and preserving its antioxidant activity inside the shrimp body.

As a first step, it was demonstrated that FITC-labeled chitosan nanoparticles had the ability to cross the intestinal barrier (Fig. 2). In addition, partition experiments with water-cod liver oil showed that the α-tocopherol is not released from the nanocapsules in a hydrophobic (lipidic) environment after 24 h. It is important to point out that while cod liver oil can partially mimic the lipophilic conditions found in the hepatopancreas, it cannot replicate them in an in vivo environment. A biological system involves digestive enzymes or pH variations, which is why this medium fails to reflect the complex interactions between the nanoparticles and the hepatopancreas.

Dietary supplementation with chitosan-nanoencapsulated α-tocopherol induced a quadratic transcriptional response in MnSOD, CAT, GPx, and GST expression, with maximal upregulation at intermediate doses (2–3 mg kg⁻¹). This biphasic pattern is consistent with hormetic-like responses, in which moderate redox modulation enhances endogenous antioxidant defenses, whereas excessive supplementation attenuates adaptive signaling (Oliveira et al. 2018). Such nonlinear behavior suggests that α-tocopherol may act as a redox regulator rather than solely as a radical scavenger, modulating intracellular signaling pathways in a dose-dependent manner.

Overall, the data suggest that α-tocopherol nanoencapsulated in chitosan can exert a practical antioxidant effect up to a specific concentration (2–3 mg Kg^−1^); however, higher doses may activate cellular defense mechanisms, likely in response to the redox imbalance induced by the compound becoming prooxidant at excessive levels. This paradoxical pro-oxidant effect of high-dose vitamin E is a well-studied phenomenon known as tocopheroxyl-mediated peroxidation, in which high concentrations of α-tocopherol lead to the accumulation of unreduced tocopheroxyl radicals that initiate lipid peroxidation (Rietjens et al. 2002). While Liu et al. (2007) demonstrated that supplementing shrimp diets with vitamin E (α-tocopherol) significantly increased the activities of antioxidant enzymes, such as superoxide dismutase (SOD), catalase (CAT), and glutathione peroxidase (GPX), compared to unsupplemented controls, especially under stress conditions, including acute salinity changes, other studies have warned that supranutritional levels of α-tocopherol can depress immune response and induce oxidative stress in aquatic organisms (Ko et al. 2025).

Despite the present study lasting 2 weeks, we believe acute responses occur within a short period, as this was the study’s purpose. Furthermore, high levels of α-tocopherol during longer periods have been reported to be toxic in rats (El-Hak et al. 2019). Nevertheless, it would be interesting to test an experimental procedure over a longer period to determine how the organisms behave and test additional parameters such as lipid and protein oxidation together with the antioxidant-related enzyme activities to confirm the limits of the hormetic response, as observed in resilient organisms, a key feature in drug development (Calabrese and Mattson 2017).

In conclusion, these findings highlight the relevance of continued research on nanoparticle-based delivery systems in aquaculture nutrition. The encapsulation of bioactive compounds such as α-tocopherol in biopolymeric matrices like chitosan not only enhances their stability but also modulates their physiological responses in a dose-dependent manner. This study establishes and optimal dietary inclusion range of 2–3 mg Kg^−1^ of nanoencapsulated α-tocopherol to maximize antioxidant defenses in shrimp. Beyond improving antioxidant efficiency, nanoencapsulation may enable precise redox regulation, opening new perspectives for functional feed design. Future research should explore the encapsulation of additional bioactive additives and evaluate long-term physiological and production outcomes to fully assess the translational potential of nanonutraceutical strategies in aquatic species.

## Data Availability

No datasets were generated or analysed during the current study.
